# Characterization of Ebola Virus–Associated Eye Disease

**DOI:** 10.1001/jamanetworkopen.2020.32216

**Published:** 2021-01-05

**Authors:** Allen O. Eghrari, Rachel J. Bishop, Robin D. Ross, Bionca Davis, Jemma Larbelee, Fred Amegashie, Robert F. Dolo, S. Grace Prakalapakorn, Catherine Gaisie, Catherine Gargu, Yassah Sosu, Jennie Sackor, Precious Z. Cooper, Augustine Wallace, Ruth Nyain, Maima Gray, Famatta Kamara, Bryn Burkholder, Christopher J. Brady, Vincent Ray, Kirstin L. Tawse, Ian Yeung, James D. Neaton, Elizabeth S. Higgs, H. Clifford Lane, Cavan Reilly, Michael C. Sneller, Mosoka P. Fallah

**Affiliations:** 1 Wilmer Eye Institute at Johns Hopkins, Baltimore, Maryland; 2 National Institutes of Health, Bethesda, Maryland; 3 Global Retina Institute, Scottsdale, Arizona; 4 Division of Biostatistics, University of Minnesota, Minneapolis; 5 Redemption Hospital, Monrovia, Liberia; 6 Liberian Ministry of Health, Monrovia, Liberia; 7 New Sight Eye Centre, Paynesville, Liberia; 8 Department of Ophthalmology, Duke University School of Medicine, Durham, North Carolina; 9 Partnership for Research on Ebola Virus in Liberia, Monrovia, Liberia; 10 University of Vermont School of Medicine, Burlington; 11 Department of Ophthalmology, California Pacific Medical Center, San Francisco; 12 Department of Ophthalmology, Kaiser Permanente, Denver, Colorado; 13 National Public Health Institute of Liberia, Monrovia, Liberia

## Abstract

**Importance:**

Survivors of Ebola virus disease (EVD) may experience ocular sequelae. Comparison with antibody-negative individuals from the local population is required to characterize the disease.

**Objective:**

To assess features of ophthalmic disease specific to EVD.

**Design, Setting, and Participants:**

This baseline cross-sectional analysis of survivors of EVD and their close contacts was conducted within PREVAIL III, a 5-year, longitudinal cohort study. Participants who enrolled at John F. Kennedy Medical Center in Liberia, West Africa from June 2015 to March 2016 were included in this analysis. Close contacts were defined as household members or sex partners of survivors of EVD. Data were analyzed from July 2016 to July 2020.

**Exposures:**

All participants, both survivors and close contacts, underwent testing of IgG antibody levels against Ebola virus surface glycoprotein.

**Main Outcomes and Measures:**

Ocular symptoms, anterior and posterior ophthalmologic examination findings, and optical coherence tomography images were compared between antibody-positive survivors and antibody-negative close contacts.

**Results:**

A total of 564 antibody-positive survivors (320 [56.7%] female; mean [SD] age, 30.3 [14.0] years) and 635 antibody-negative close contacts (347 [54.6%] female; mean [SD] age, 25.8 [15.5] years) were enrolled in this study. Survivors were more likely to demonstrate color vision deficit (28.9% vs 19.0%, odds ratio [OR], 1.6; 95% CI, 1.2-2.1) and lower intraocular pressure (12.4 vs 13.5 mm Hg; mean difference, −1.2 mm Hg; 95% CI, −1.6 to −0.8 mm Hg) compared with close contacts. Dilated fundus examination revealed a higher percentage of vitreous cells (7.8% vs 0.5%; OR, 16.6; 95% CI, 5.0-55.2) and macular scars (4.6% vs 1.6%; OR, 2.8; 95% CI, 1.4-5.5) in survivors than in close contacts. Uveitis was present in 26.4% of survivors and 12.1% of close contacts (OR, 2.4; 95% CI, 1.8-3.2). Among all participants with uveitis, survivors were more likely than close contacts to have intermediate uveitis (34.2% vs 6.5% of all cases; OR, 7.8; 95% CI, 3.1-19.7) and had thicker mean central subfield thickness on optical coherence tomography (222 vs 212 μm; mean difference, 14.4 μm; 95% CI, 1.9-26.9 μm).

**Conclusions and Relevance:**

In this cross-sectional study, survivors of EVD had a distinct spectrum of ocular and neuro-ophthalmologic findings compared with close contacts that potentially require medical and surgical treatment.

## Key Points

**Question:**

Do survivors of Ebola virus disease have more eye problems than the general population?

**Findings:**

In this cross-sectional study of 564 Ebola virus antibody–positive survivors and 635 antibody-negative close contacts, survivors had higher rates of uveitis, decreased intraocular pressure, impairment of color vision, and decreased accommodative tone. Biomicroscopic examination and optical coherence tomography revealed retinal scars and macular edema in survivors.

**Meaning:**

The findings suggest that Ebola virus disease is associated with development of a spectrum of changes throughout the visual pathway that may persist after acute infection.

## Teaser

This cross-sectional analysis of compares rates of ocular sequelae in antibody-positive survivors of Ebola virus disease and antibody-negative close contacts.

## Introduction

*Zaire ebolavirus* is a negative, single-stranded RNA virus associated with high rates of morbidity and mortality in infected humans. The West Africa epidemic of Ebola virus disease (EVD) led to approximately 11 000 deaths and is the largest epidemic of this disease to date.^1^

Ocular symptoms can occur as part of the initial presentation of EVD or after resolution of the systemic illness.^2^ Of 4 survivors with uveitis reported from the 1995 epidemic in Zaire (now named Democratic Republic of the Congo), all ocular symptoms occurred more than 40 days after onset of EVD symptoms, with the latest occurring 72 days after EVD onset.^3^ In a patient with uveitis occurring after systemic convalescence during the West Africa epidemic, viable Ebola virus was detected in the aqueous humor 9 weeks after viremia resolved.^4^ Polymerase chain reaction testing of tear samples has detected virus during active infection but not at 3 months.^4^ To date, viral RNA has not been identified in samples of aqueous humor obtained from survivors undergoing cataract surgery more than 1 year after infection,^5^ although data are limited because eyes without inflammation are preferentially selected for surgery.

Ophthalmic sequelae are highly represented in cohorts of survivors who have presented to facilities for care after EVD has resolved.^2,6,7,8^ However, without serologically confirmed individuals from a control population, it is unclear to what extent the specific symptoms and pathologic changes experienced by survivors of EVD are attributable to the virus.

A research partnership was established between the Liberia Ministry of Health and Social Welfare and the US National Institute of Allergy and Infectious Diseases, National Institutes of Health titled the Partnership for Research on Ebola Virus in Liberia (PREVAIL), to build a sustainable local framework for research and clinical care in the context of EVD and other infectious diseases. PREVAIL III, a natural history study of survivors of EVD, provided longitudinal follow-up of survivors and their close contacts for 5 years.

A previous report from PREVAIL III^2^ revealed that uveitis was present in 26% of survivors of EVD and 12% of their close contacts at baseline. That report highlighted the importance of a comparison group from the population to delineate pathology specific to survivors of EVD. Because prior case reports have described Ebola virus–associated uveitis,^3^ we hypothesized that a higher rate of uveitis would be present in survivors of EVD compared with their close contacts. However, because investigators recognize that Ebola virus–associated eye disease may include findings beyond uveitis, the objective of the eye substudy of PREVAIL III was to compare rates of ophthalmic pathology in survivors of EVD and their close contacts over a 5-year period.

## Methods

This baseline cross-sectional study included survivors of EVD and their close contacts from the PREVAIL III eye substudy. The study was approved by the institutional review board and ethics committee at the National Institute of Allergy and Infectious Diseases, National Institutes of Health, and the Liberian National Research Ethics Board. All patients participated in a detailed consent briefing and provided written informed consent. This study followed the Strengthening the Reporting of Observational Studies in Epidemiology (STROBE) reporting guideline.^9^

### Participants

Recruitment occurred at 3 sites with use of lists maintained by the Liberia Ministry of Health and Social Welfare of survivors diagnosed with EVD at an Ebola treatment unit. All individuals included in these lists were eligible to be enrolled as survivors. Close contacts were defined as household members, friends, or neighbors of survivors at the time of diagnosis or after recovery from EVD and sexual partners of the survivors after discharge from the treatment facility. All survivors and their close contacts registered in the PREVAIL III parent study from June 2015 to March 2016 were eligible for an eye substudy, which included an ophthalmic evaluation at the PREVAIL eye clinic at John F. Kennedy Medical Center in Monrovia, Liberia, and yearly ophthalmic follow-up examinations from June 2015 to June 2020. Examiners did not have direct access to enrollment status of participants.

### Study Design

Because some individuals included in survivor lists did not have polymerase chain reaction–documented evidence of infection, all survivors of EVD in PREVAIL III underwent serologic confirmation of prior infection by measurement of anti–Ebola virus antibodies using the Filovirus Non-Clinical Animal Group assay according to methods described elsewhere.^10^ Seropositivity was defined as having an Ebola virus glycoprotein IgG antibody titer of 548 U/mL or higher on enzyme-linked immunosorbent assay, and seronegativity was defined as having an antibody titer below 548 U/mL on enzyme-linked immunosorbent assay.^2,10^ Close contacts also underwent testing for anti–Ebola virus antibodies to confirm group classification in this study.

Study evaluations included an ocular history and current symptoms, comprehensive ophthalmic examinations, and ophthalmic imaging. Ocular examination included visual acuity assessment with an ETDRS Tumbling E chart, color vision assessment with 14 Ishihara color plates, noncycloplegic autorefraction, best-corrected spherical equivalent visual acuity with phoropter, confrontational visual fields, tests of alignment and ocular motility, pupil examination, intraocular pressure assessment with rebound tonometry (Icare) and disposable probes, slitlamp biomicroscopic examination of the anterior segment, and dilated fundus examination with indirect fundoscopy. Optical coherence tomography (OCT) of the optic nerve and macula was performed for all participants older than 4 years with a Zeiss Cirrus 5000 OCT device (Carl Zeiss Meditec Inc). All participants were examined by a licensed ophthalmologist.

Uveitis was diagnosed if 1 or more of the following findings were discovered on eye examination: keratin precipitates, anterior chamber cells or flare, hypopyon, posterior synechiae, vitreous cells or haze, retinal scar (macular or peripheral), or vascular sheathing without hypertensive retinopathy.

Each OCT image was assessed by the examining ophthalmologist (A.O.E., R.J.B., R.D.R., J.L., F.A., S.G.P., B.B., C.J.B., V.R., K.L.T., and I.Y.) at the time of evaluation for the presence of epiretinal membrane, intraretinal fluid, or vitreous opacities. Given that no standardized images existed for this population, a new set was created to delineate 3 levels of vitreous opacities: none to minimal, mild, and moderate to severe. Levels were based on the spectrum of opacities observed in the first 20 images. All ophthalmologists reviewed the standardized images obtained at the beginning of the study. These are included in the eFigure in the Supplement.

Data obtained from the parent PREVAIL III study included date of infection with EVD, laboratory test results (including testing for presence of Ebola RNA in semen samples in a cohort of male survivors), and comprehensive medical examination findings. Standard-of-care treatment was initiated for any ophthalmic disorders requiring medical therapy. In addition to regular study visits, all participants requiring medical treatment received follow-up care as clinically indicated.

### Statistical Analysis

Data were analyzed from July 2016 to July 2020. All statistical analyses were performed using SAS, version 9.3 (SAS Institute Inc) or R, version 3.2.3 (R Project for Statistical Computing). Statistical comparison was conducted between Ebola antibody–positive survivors and antibody-negative close contacts, between those with and without uveitis among antibody-positive survivors of EVD and antibody-negative close contacts, and between male survivors with and without semen sample(s) positive for Ebola virus RNA.

We used generalized estimating equations with a logistic link for dichotomous variables, such as demographic details or questionnaire answers. For continuous variables, logarithmic transformation was applied before generalized estimating equations were used for parameter estimation. All generalized estimating equation models assumed an independence correlation structure, and robust variance estimators were used for construction of 95% CIs and computation of *P* values. Random effects were associated with groups of related survivors and close contacts, and models were adjusted for sex and age. The Fisher exact test was used for values of 0. All *P* values cited are 2-sided and not adjusted for the multiple outcomes considered, with a priori level of significance at *P* = .05. We chose this approach given the effect of such adjustment on increasing the risk of a type II error when comparing features that occurred in a low proportion of survivors and close contacts. Therefore, *P* values that marginally meet the threshold for statistical significance should be interpreted with caution. We include effect estimates as odds ratios (ORs) or adjusted mean differences and 95% CIs when comparing rates of findings between groups.

## Results

### Demographic Characteristics

**Figure 1.**
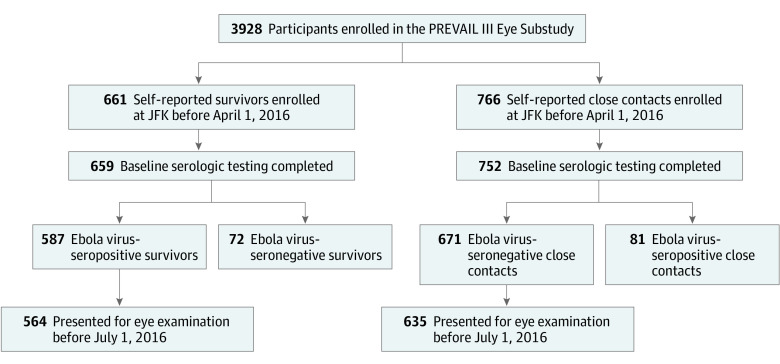
Flowchart of Enrollment in the PREVAIL III Eye Substudy Serologically confirmed status of both survivors of Ebola virus disease and their close contacts allowed for more accurate comparison across groups and determination of Ebola-specific pathology. JFK indicates John F. Kennedy Medical Center.

Figure 1 shows the enrollment of 3928 participants in the PREVAIL III study. Of these individuals, 564 antibody-positive survivors of EVD and 635 antibody-negative close contacts were enrolled in the study at the John F. Kennedy Medical Center study site and underwent detailed baseline ophthalmic examinations during the target period. This group of participants comprised the PREVAIL III eye substudy cohort from which data are presented. Of note, the self-reported status of approximately 1 in 10 individuals who enrolled in each group did not match the serologic status; these participants were excluded from analysis.

Baseline demographic and clinical characteristics of the study population are summarized in eTable 1 in the Supplement. The mean (SD) age for survivors was 30.3 (14.0) years, and the mean (SD) age for close contacts was 25.8 (15.5) years at the time of enrollment. A total of 320 survivors (56.7%) and 347 close contacts (54.6%) were women. The mean (SD) interval between enrollment and eye examination was 100 (94.0) days among survivors and 64 (62.5) days among close contacts (mean difference, 38.8 days; 95% CI, 28.7-48.9 days).

At the time of examination, survivors more frequently reported trouble seeing (49.1% vs 36.1%; OR, 1.4; 95% CI, 1.1-1.9), sensitivity to light (49.1% vs 40.1%; OR, 1.4; 95% CI, 1.1-1.7), and eye redness (25.6% vs 18.4%; OR, 1.5; 95% CI, 1.1-1.9). The median interval from symptom onset to enrollment was 338 days (IQR, 293-385 days) among survivors without uveitis and 335 days (IQR, 286-367 days) among survivors with uveitis (mean difference, 3.4 days; 95% CI, –16.4 to 9.6 days).

### Visual Function Testing

Table 1 summarizes ocular examination findings in survivors of EVD and their close contacts. Both survivors and close contacts demonstrated median best-corrected spherical equivalent visual acuity of 20/20. A higher percentage of survivors demonstrated color vision deficit (28.9% vs 19.0%; OR, 1.6; 95% CI, 1.2-2.1). Accommodative tone, determined by the Prince rule after refractive correction, suggested a further near point in survivors, with the top quartile of survivors achieving a near point of less than 20 cm compared with 15 cm in close contacts (mean difference, –1.8; 95% CI, –3.0 to –0.5). Median intraocular pressure was significantly lower in survivors (12.4 mm Hg [IQR, 10.4-14.5] vs 13.5 mm Hg [IQR, 11.5-16.0]; mean difference, –1.2 mm Hg ; 95% CI, –1.6 to –0.8 mm Hg).

**Table 1.** Clinical Examination Findings Among Survivors of Ebola Virus Disease and Close Contacts

Finding	Survivors (n = 564)		Close contacts (n = 635)		*P* value	Effect estimate (95% CI)^a^
	Value	With data, No.	Value	With data, No.		
**Tests of visual function**						
Presenting visual acuity, denominator of the Snellen fraction						
Median (IQR)	22.4 (20.0 to 30.0)	556	22.4 (20.0 to 25.0)	611	.28	−25.1 (−70.9 to 20.8)
Mean (SD)	44.1 (128.0)		53.4 (372.0)		NA	NA
BCVA, denominator of the Snellen fraction						
Median (IQR)	20.0 (20.0 to 25.0)	556	20.0 (20.0 to 25.0)	611	.30	−24.1 (−69.7 to 21.5)
Mean (SD)	38.4 (121.0)		49.4 (373.0)		NA	NA
Pinhole visual acuity, denominator of the Snellen fraction						
Median (IQR)	28.3 (20.0 to 38.7)	140	25.1 (25.0 to 38.9)	106	.09	−32.7 (−70.6 to 5.2)
Mean (SD)	44.3 (69.4)		80.3 (210.0)		NA	NA
Near point, cm						
Median (IQR)	20.0 (20.0 to 25.0)	535	20.0 (15.0 to 25.0)	624	.007	−1.8 (−3.0 to −0.5)
Mean (SD)	25.8 (14.3)		24.4 (14.9)		NA	NA
Intraocular pressure, mm Hg						
Median (IQR)	12.4 (10.4 to 14.5)	557	13.5 (11.5 to 16.0)	632	*<*.001	−1.2 (−1.6 to −0.8)
Mean (SD)	12.7 (3.5)		14 (3.5)		NA	NA
Spherical equivalent, D						
Median (IQR)	0.1 (−0.3 to 0.4)	526	0.1 (−0.3 to 0.4)	618	.67	0.0 (−0.1 to 0.1)
Mean (SD)	0.03 (0.9)		0.0 (0.9)		NA	NA
Confrontational visual field deficit, No. (%)	30.0 (5.3)	564	22.0 (3.5)	635	.55	1.2 (0.7 to 2.2)
Color vision deficit, No. (%)						
<14/14	156.0 (28.9)	539	112.0 (19.0)	591	.002	1.6 (1.2 to 2.1)
<12/14	61.0 (11.4)	537	33.0 (5.6)	590	.006	1.8 (1.2 to 2.9)
Afferent pupillary defect, No. (%)	13.0 (2.3)	561	11.0 (1.7)	633	.91	1.0 (0.4 to 2.2)
**Biomicroscopic examination**						
Keratic precipitates, No. (%)^b^	34.0 (6.0)	564	17.0 (2.7)	635	.01	2.2 (1.2 to 4.0)
Anterior chamber cells, No. (%)^b^	22.0 (3.9)	564	9.0 (1.4)	635	.008	3.1 (1.3 to 7.0)
Posterior synechiae, No. (%)^b^	24.0 (4.3)	564	3.0 (0.5)	635	*<*.001	10.0 (2.8 to 36.6)
Cataract, No. (%)	78.0 (13.8)	564	81.0 (12.8)	635	.10	0.7 (0.4 to 1.1)
Cataract with BCSEVA <20/40, No. (%)	22.0 (3.9)	564	16.0 (2.5)	635	.73	1.1 (0.6 to 2.2)
Age, No. (%)						
*≤*40 y	10.0 (1.8)	542	2.0 (0.3)	603	.02	NA
>40 y^c^	12.0 (2.2)	558	14.0 (2.2)	634	*>*.99	NA
Vitreous cells, No. (%)^b^	44.0 (7.8)	564	3.0 (0.5)	635	*<*.001	16.7 (5.0 to 55.3)
Optic nerve swelling, No. (%)	4.0 (0.7)	564	3.0 (0.5)	635	.57	1.6 (0.3 to 7.2)
Macular edema, No. (%)	5.0 (0.9)	564	0	635	.02	NA
Retinal scar, No. (%)^b^						
Macula	26.0 (4.6)	564	10.0 (1.6)	635	.004	2.8 (1.4 to 5.5)
Periphery	56.0 (9.9)	564	37.0 (5.8)	635	.02	1.7 (1.1 to 2.5)
Uveitis of any type, No. (%)	149.0 (26.4)	564	77.0 (12.1)	635	*<*.001	2.4 (1.8 to 3.3)
Cup-disc ratio						
Median (IQR)	0.4 (0.3 to 0.5)	537	0.3 (0.2 to 0.4)	625	.04	0.02 (0 to 0.04)
Mean (SD)	0.4 (0.2)	NA	0.3 (0.2)	NA	NA	NA

Abbreviations: BCSEVA, best-corrected spherical equivalent visual acuity; IQR, interquartile range; NA, not applicable.

^a^ Effect estimates for medians (IQRs) are mean difference; data for the other rows are odds ratios. All estimates were adjusted for age, sex, uveitis, and relationships among survivors and close contacts unless otherwise noted.

^b^ Estimates were adjusted for sex, uveitis, and relationships among survivors and close contacts.

^c^ The Fisher exact test was used to test for differences.

### Biomicroscopic Examination

**Figure 2.**
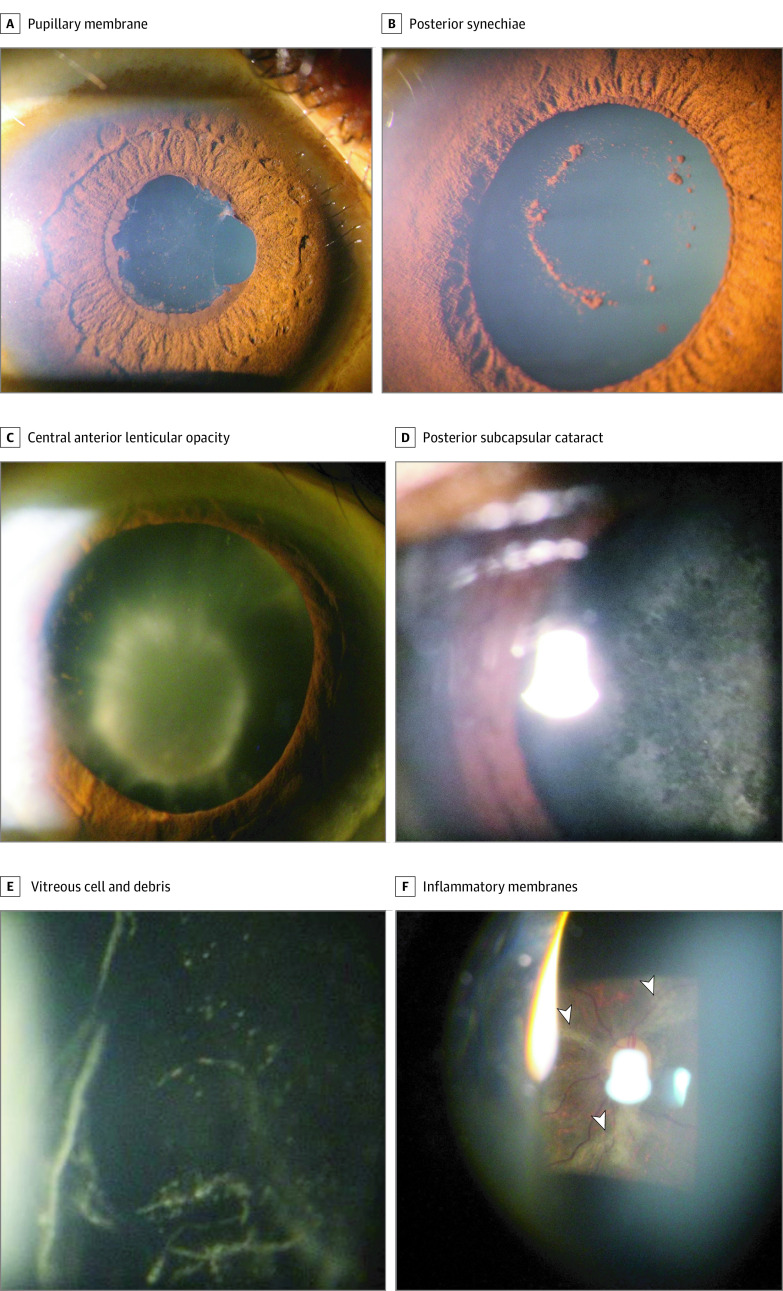
Slitlamp Biomicroscopic Examination of Eyes Affected by Ebola Virus Disease A, Posterior synechiae and pupillary membrane appeared in an eye with anterior uveitis. B, Pigment on the anterior lens capsule remained after lysis of synechiae with cycloplegic drops. C and D, Inflammatory cataracts in eyes with a history of uveitis included central, anterior lenticular opacity (C), and posterior subcapsular cataract (D). E, Vitreous cells and debris appeared in an eye with intermediate uveitis. F, As imaged through a 90-D lens, gliotic processes (arrowheads) are seen extending from the optic nerve in the eye of a survivor of Ebola virus disease.

Table 1 also reveals findings from slitlamp examination and indirect biomicroscopic examination in survivors of EVD and their close contacts. Survivors more frequently demonstrated inflammatory changes, including posterior synechiae (4.3% vs 0.5%; OR, 10.0; 95% CI, 2.7-36.6), vitreous cells (7.8% vs 0.5%; OR, 16.7; 95% CI, 5.0-55.3), and retinal scars in the macula (4.6% vs. 1.6%; OR, 2.8; 95% CI 1.4-4.5). Figure 2 shows these findings in images acquired through the slitlamp microscope.

### Uveitis

Given that uveitis may manifest with signs and symptoms specific to its cause, we sought to identify the clinical characteristics of uveitis experienced by survivors of EVD. Table 2 compares demographic characteristics, eye symptoms, tests of visual function, OCT findings, and results of diagnostic testing in both survivors of EVD and their close contacts with uveitis. Uveitis was present in 26.4% of survivors and 12.1% of close contacts (OR, 2.4; 95% CI, 1.8-3.2).

**Table 2.** Baseline Demographic Characteristics, Baseline Laboratory Measurements, and Vision Complications for Survivors of EVD and Close Contacts With Uveitis

	Survivors (n = 149)		Close contacts (n = 77)		*P* value	Effect estimate (95% CI)^a^
	Value	With data, No.	Value	With data, No.		
**Demographic characteristics**						
Age, y						
Median (IQR)	32 (24 to 43)	149	34 (25 to 44)	77	.50	1.0 (1.0 to 1.0)
Mean (SD)	33.2 (14.1)		34.6 (15.5)		NA	NA
Female, No. (%)	78 (52.3)	149	41 (53.2)	77	.91	1.0 (0.6 to 1.7)
Eye symptoms, No. (%)						
Trouble seeing	100 (67.6)	148	41 (53.2)	77	.02	2.1 (1.1 to 3.9)
Pain in eye	68 (45.9)	148	33 (42.9)	77	.64	1.1 (0.7 to 2.0)
Sensitivity to light	82 (55.4)	148	37 (48.1)	77	.3	1.4 (0.8 to 2.4)
Redness	39 (26.4)	148	19 (24.7)	77	.75	1.1 (0.6 to 2.1)
Discharge	29 (22.1)	131	20 (31.2)	64	.12	0.6 (0.3 to 1.1)
**Tests of visual function**						
Visual acuity, denominator of the Snellen fraction						
Median (IQR)	25.0 (20.0 to 44.7)	148	25.0 (20.0 to 35.8)	77	.30	−115.5 (−334.2 to 103.3)
Mean (SD)	61.0 (119.0)		181.0 (1021.0)		NA	NA
Best-corrected visual acuity, denominator of the Snellen fraction						
Median (IQR)	22.4 (20.0 to 32.8)	148	25.0 (20.0 to 28.3)	77	.30	−115.5 (−333.9 to 102.9)
Mean (SD)	49.6 (98.0)		168.0 (1018.0)		NA	NA
Pinhole visual acuity, denominator of the Snellen fraction						
Median (IQR)	35.5 (22.4 to 50.0)	52	26.8 (25.0 to 38.9)	26	.62	−14.8 (−74.0 to 44.4)
Mean (SD)	56.1 (81.3)		75.9 (149)		NA	NA
Color vision, Ishihara color plates						
Median (IQR)	14 (13.0 to 14.0)	141	14.0 (13.7 to 14.0)	67	.01	−0.8 (−1.4 to −0.2)
Mean (SD)	12.7 (3.30)		13.5 (1.47)		NA	NA
Intraocular pressure, mm Hg						
Median (IQR)	12.0 (10.0 to 14.0)	149	13.5 (11 to 16.2)	76	*<*.001	−2.1 (−3.1 to −1.1)
Mean (SD)	12.2 (3.03)		14.2 (4.1)		NA	NA
**Ocular coherence tomography**						
Vitreous opacities, No. (%)	61 (47.3)	129	21 (30.9)	68	.02	2.1 (1.1 to 3.8)
Intraretinal fluid, No. (%)	13 (10.2)	127	1 (1.5)	68	.06	7.8 (0.9 to 65.2)
Epiretinal membrane, No. (%)	9 (11.7)	77	14 (20.6)	68	.16	0.5 (0.2 to 1.3)
Central subfield thickness, μm						
Median (IQR)	222 (210 to 236)	85	212 (200 to 219)	29	.02	14.4 (1.9 to 26.9)
Mean (SD)	222 (23.3)		209 (27.9)		NA	NA
**Diagnostic testing**						
Anterior uveitis, No. (%)	57 (38.3)	149	26 (33.8)	77	.58	1.2 (0.7 to 2.2)
Intermediate uveitis, No. (%)	51 (34.2)	149	5 (6.5)	77	*<*.001	7.8 (3.1 to 19.7)
Posterior uveitis, No. (%)	69 (46.3)	149	50 (64.9)	77	.009	0.5 (0.3 to 0.8)
Panuveitis, No. (%)	8 (5.4)	149	1 (1.3)	77	.16	4.5 (0.6 to 35.9)
HIV seropositive, No. (%)^b^	0	141	5 (7.1)	70	.004	0 (0 to 0.5)
Syphilis seropositive, No. (%)	7 (5.0)	141	3 (4.3)	70	.70	1.3 (0.3 to 5.0)

Abbreviations: EVD, Ebola virus disease; IQR, interquartile range.

^a^ Effect estimates for medians (IQRs) are mean difference; data for the other rows are odds ratios. All estimates were adjusted for age, sex, and relationships among survivors and close contacts.

^b^ The Fisher exact test was used to test for difference.

Compared with uveitis in eyes of close contacts, uveitis in the eyes of survivors of EVD was more likely to manifest as intermediate (34.2% vs 6.5% of total cases of uveitis; OR, 7.8; 95% CI, 3.1-19.7), decreased median intraocular pressure (12.0 mm Hg [IQR, 10.0-14.0] vs 13.5 mm Hg [IQR, 11.0-16.2]; mean difference, –2.1 mm Hg; 95% CI, –3.1 to –1.1 mm Hg), and increased median central subfield thickness of the macula as measured by OCT imaging (222 μm [IQR, 210-236 μm] vs 212 μm [IQR, 200-219 μm]; mean difference, 14.4 μm; 95% CI, 1.9-26.9 μm).

**Figure 3.**
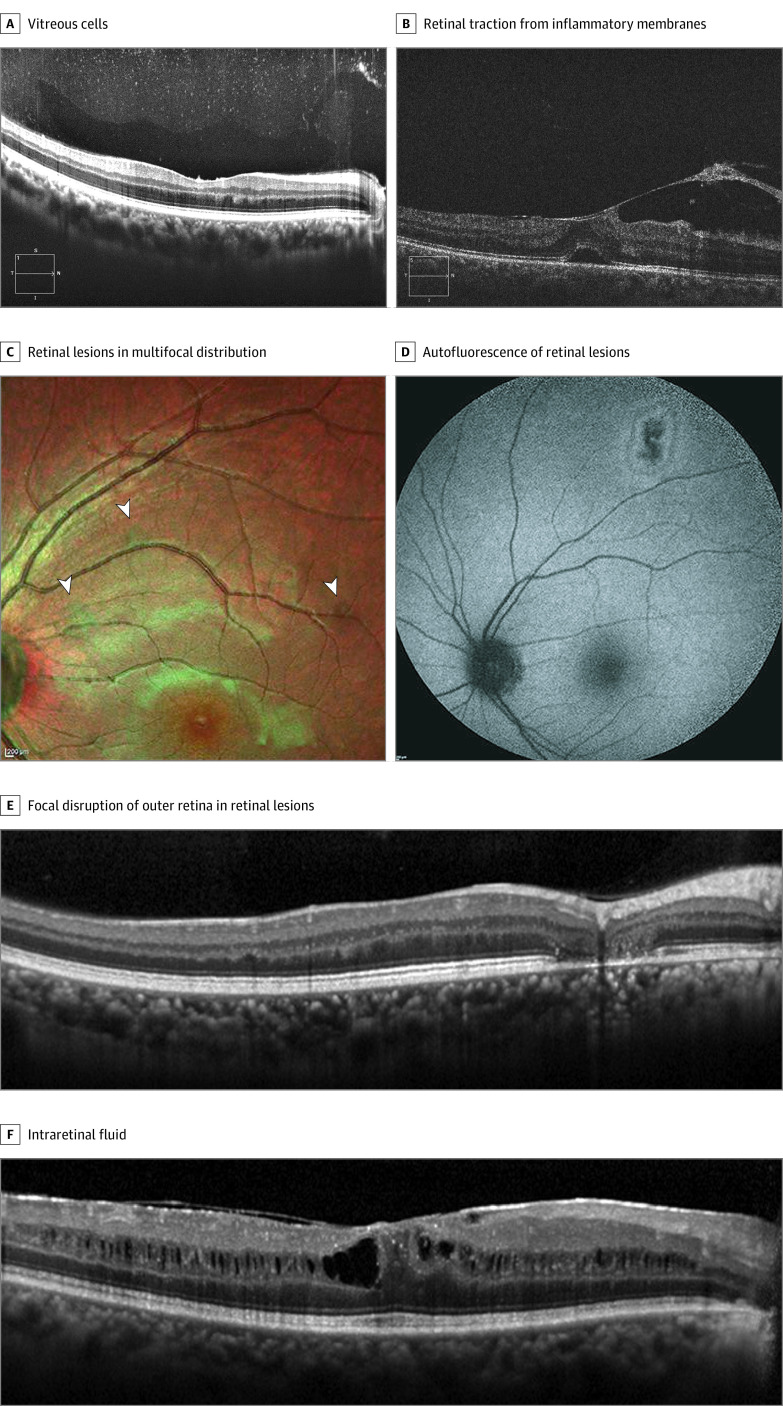
Optical Coherence Tomography of Eyes of Survivors of Ebola Virus Disease A, Vitreous cells appeared as punctate opacities distributed through the vitreous gel. B, Vitreomacular traction appeared in a young male survivor with inflammatory membrane formation. C and D, Retinal lesions showed a multifocal distribution and on both multicolor (arrowheads) (C) and autofluorescence (D) images. E, These lesions characteristically affected the outer retina. F, Macular edema appeared in a diffuse pattern throughout the inner nuclear layer.

Optical coherence tomography was performed for 542 of 564 survivors of EVD (96.1%) and 619 of 635 close contacts (97.5%). Figure 3 shows qualitative characteristics of ocular pathology identified by OCT. Of note, retinal lesions were multifocal and primarily induced disruption of the outer retina, sparing the choroid.

eTable 2 in the Supplement compares clinical characteristics of survivors of EVD with and without uveitis. Survivors with uveitis had a longer median stay in the Ebola treatment unit (18.5 days [IQR, 12.3-21.8 days] vs 15.0 days [IQR, 11.0-19.0 days]; mean difference, 2.7 days; 95% CI, 1.2-4.2 days), were more likely to report eye pain (45.9% vs 30.5%; OR, 2.06; 95% CI, 1.4-3.1), and had lower intraocular pressure (12.0 mm Hg [IQR, 10.0-14.0 mm Hg] vs 12.5 mm Hg [IQR, 10.5-14.7 mm Hg]; mean difference, −0.7 mm Hg; 95% CI, −1.4 to −0.04 mm Hg).

## Discussion

In this baseline cross-sectional study of the 5-year PREVAIL III longitudinal cohort study of antibody-positive survivors of EVD and their antibody-negative close contacts in Liberia, West Africa, we aimed to classify ocular changes associated with EVD. The data reveal a spectrum of intraocular and neuro-ophthalmologic pathology.

The need for a comparison group in this setting is supported by the high percentage of close contacts with ocular symptoms. For example, approximately 2 of 5 close contacts reported sensitivity to light. We found that many participants in both groups experienced significant ocular surface disease, making a comparison group particularly important. Of note, the serologic testing results of approximately 1 in 10 individuals who enrolled in each group excluded them from analysis (eg, close contacts who tested positive for antibodies), supporting the need for serologically proven status.

Visual acuity remained intact as measured in most survivors; however, the ability to read characters reflects resolution within the central visual field, and retinal scars were distributed without a propensity for the fovea. Clinically significant color vision loss, defined as at least 3 missed Ishihara plates, was almost twice as likely to occur in survivors of EVD as in close contacts. In addition, at the 75th percentile in each group, survivors demonstrated a decrease of approximately 1 D in accommodative tone, suggesting that even a year after discharge from the Ebola treatment unit, many survivors experienced decreased ability to engage in close work. Together, these signs point to multifaceted visual deficits that may involve pathology distributed throughout the central nervous system.

This study draws on 1 of the largest samples of OCT from a West African population. The data revealed a pattern of inflammatory changes, including epiretinal membrane, intraretinal fluid, and retinal scars, in survivors of EVD. The specific pattern of multifocal retinal lesions that affects the outer retina in patients with EVD has been previously reported^11^; these characteristics were subsequently reported in a cohort in Sierra Leone^12^ and qualitatively investigated by means of multimodal imaging.^13^ Data from subsequent years will help to clarify changes in the morphologic features of retinal lesions over time.

The imaging data also highlight the need to expand current normative standards of retinal anatomy. Compared with normative OCT data used worldwide, the central macula of many study participants appeared thin because the threshold of normal central subfield thickness is defined by the OCT device manufacturer as a range of 220.5 to 298 μm.^14^ The mean thickness of the central macula in study participants approximated the lower limit, suggesting that such guidelines require revision to be fully used by diverse patient populations.

In Table 1, a subanalysis highlights the experience of survivors younger than 40 years with cataracts that result in visual impairment (best-corrected spherical equivalent visual acuity <20/40). This group was specifically assessed because the cause of cataracts varies during the life span, with age-related cataracts in West Africa primarily affecting individuals older than 40 years.^15^ Although this finding is consistent with our clinical experience in treatment of inflammatory cataracts in individuals aged 16 years or older, it must be interpreted with caution because of the low rate of cataract formation in each group and the marginal value of significance. Findings from future studies may provide more information on specific risk factors or features of cataracts that correlate more strongly with disease status.

Clinically, these findings suggest that post-EVD care may require both medical and surgical approaches to therapy. Posterior synechiae, pupillary membranes, cataracts, epiretinal membranes, and vitreoretinal traction are aspects of clinical phenotypes for which intraocular surgery may be required to restore vision. Reports of outcomes compared with those in a population-based comparison group would help clarify the utility of surgery in this context. To date, findings from case series^5^ of cataract surgeries in survivors of EVD have been encouraging. Assessments of rates of viral persistence and cataract surgery outcomes are the subject of the PREVAIL VII study.^16^

Together, the data support strengthening of health care systems to diagnose and treat sequelae of EVD. In addition, the results of this cooperative effort by governments, organizations, individuals, and the local community highlight the importance of collaboration and the feasibility of executing a large ophthalmic study in the setting of an emerging infectious disease.

### Limitations

This study has limitations. As a baseline cross-sectional analysis, a limitation of this study is that the data captured a relatively narrow window in time for survivors of EVD. Longitudinal evaluation of a set of participants over 5 years would provide insight into the incidence of new findings and resolution of current symptoms. Moreover, additional data acquired immediately after acute infection would assist in clarifying the timeline of findings nearer the onset of symptoms.

Within the group of close contacts, an additional limitation is the possible enrichment of ocular pathology. Limited local availability of eye care services may motivate individuals with the need for eye care to enroll and undergo examinations. This may result in a decreased ability to distinguish findings specific to survivors of EVD and would result in a conservative estimate of the total range of pathology associated with EVD.

In addition, as an exploratory analysis of a novel clinical presentation performed during an outbreak of an emerging infectious disease, this study was unable to completely mask examiners with regard to the goals of the study or the disease status of all individuals. Although examiners did not have direct access to enrollment status of participants, other study personnel had access to this information, and formal attempts to mask examiners to participant enrollment status were not conducted.

## Conclusions

This baseline cross-sectional study of survivors of EVD in the longitudinal PREVAIL III study revealed a spectrum of changes within the visual pathway and clarified that uveitis was a part of a multifaceted disease process. The detection of these changes approximately 1 year after onset of symptoms highlights the importance of sustained efforts to provide care for survivors of EVD even after acute infection has resolved.

## Notes

Supplement. **eTable 1.** Baseline Demographic and Clinical Characteristics of Survivors and Close Contacts **eTable 2.** Clinical Characteristics of Ebola Virus Disease Survivors With and Without Uveitis **eFigure.** Standard Optical Coherence Tomography Images Viewed by Examiners to Guide Subjective Grading of the Level of Vitreous Opacities in the PREVAIL III Eye Substudy
