# Supplementary material for: Characterization of Ebola Virus–Associated Eye Disease
Source: JAMA Netw Open. 2021 Jan 5;4(1):e2032216. doi: 10.1001/jamanetworkopen.2020.32216 (PMC7786253; doi:10.1001/jamanetworkopen.2020.32216)
Supplement: Supplement. — eTable 1. Baseline Demographic and Clinical Characteristics of Survivors and Close Contacts eTable 2. Clinical Characteristics of Ebola Virus Disease Survivors With and Without Uveitis eFigure. Standard Optical Coherence Tomography Images Viewed by Examiners to Guide Subjective Grading of the Level of Vitreous Opacities in the PREVAIL III Eye Substudy [file jamanetwopen-e2032216-s001.pdf]

Supplementary Online Content

Eghrari AO, Bishop RJ, Ross RD, et al. Characterization of Ebola virus–associated eye disease. *JAMA Netw Open.* 2020;4(1):e2032216. doi:10.1001/jamanetworkopen.2020.32216

eTable 1. Baseline Demographic and Clinical Characteristics of Survivors and Close Contacts

eTable 2. Clinical Characteristics of Ebola Virus Disease Survivors With and Without Uveitis

eFigure. Standard Optical Coherence Tomography Images Viewed by Examiners to Guide Subjective Grading of the Level of Vitreous Opacities in the PREVAIL III Eye Substudy

This supplementary material has been provided by the authors to give readers additional information about their work.

© 2021 Eghrari AO et al. JAMA Network Open.

eTable 1: Baseline demographic and clinical characteristics of survivors and close contacts						
	Survivors (N=564)	N with data	Close Contacts (N=635)	N with data	p-value	Odds Ratio (95% CI)
**Demographics**						
Female	320 (56.7%)	564	347 (54.6%)	635	0.49	1.09 (0.85, 1.39)
Age (years) Mean (SD)	30.3 (14)	564	25.8 (15.5)	635	< 0.001	1.02 (1.01,1.03)
**Age category**						
< 18 years	101 (17.9%)	-	232 (36.5%)	-	-	-
18-45 years	375 (66.5%)	-	321 (50.6%)	-	< 0.001	-
> 45 years	88 (15.6%)	-	82 (12.9%)	-	-	-
HIV seropositive	9 (1.7%)	515	12 (2.4%)	505	0.43	0.71 (0.3, 1.67)
Syphilis seropositive	23 (4.5%)	515	20 (4%)	505	0.8	1.08 (0.58, 2.03)
**Previous eye problems**						
Eye problems	400 (71%)	563	469 (73.9%)	635	0.02	0.72 (0.55, 0.95)
Surgery	7 (1.2%)	563	5 (0.8%)	635	0.58	1.39 (0.43, 4.51)
Infection	168 (35.6%)	472	169 (38.8%)	436	0.26	0.85 (0.64, 1.13)
Trauma	95 (20.1%)	472	126 (28.9%)	436	0.006	0.64 (0.47, 0.88)
Medication use	216 (45.9%)	471	188 (43.1%)	436	0.57	1.08 (0.83, 1.41)
**Complaints at baseline**						
Trouble seeing	276 (49.1%)	562	229 (36.1%)	635	0.008	1.45 (1.1, 1.9)
Eye pain	194 (34.6%)	561	184 (29%)	634	0.11	1.23 (0.95, 1.59)
Light sensitivity	276 (49.1%)	562	254 (40.1%)	634	0.01	1.36 (1.06, 1.74)
Redness	144 (25.6%)	562	117 (18.4%)	635	0.008	1.46 (1.1, 1.93)
Discharge	102 (21.7%)	471	104 (23.9%)	436	0.43	0.88 (0.63, 1.21)
			**Enrollment**			**Mean difference**
Mean Enrollment to eye exam, days (SD)	100 (94)	564	64 (62.5)	635	< 0.001	38.8 (28.66, 48.93)

© 2021 Eghrari AO et al. JAMA Network Open.

Estimates are adjusted for age, sex, and relationships among survivors and close contacts. SD: Standard Deviation

© 2021 Eghrari AO et al. JAMA Network Open.

eTable 2. Clinical characteristics of Ebola virus disease survivors with and without uveitis			
	**Uveitis (N=149)**	**No uveitis (N=415)**	**Effect Estimate (95% CI)**
	**Demographics**		**Odds ratio**
**Age (yrs) Median (IQR)**	32 (24-43)	28 (19.5-38)	1.02 (1.01, 1.03)
**Female**	78 (52.3%)	242 (58.3%)	0.78 (0.54, 1.15)
	**Antibody level (EU)**		**Mean Difference**
**Median (IQR)**	19910 (13950-29960)	19420 (12000-34350)	15.42 (-4456.25, 4487.09)
**Mean (SD)**	25870 (23672)	26970 (23996) 28350 (25989)	
	**Ebola Treatment Unit Stay (days)**		**Mean difference**
**Median (IQR)**	18.5 (12.25-21.75)	15 (11-19)	2.69 (1.15, 4.24)
**Mean (SD)**	17.97 (7.7)	15.32 (6.98)	
	**Symptom onset to enrollment (days)**		**Mean difference**
**Median (IQR)**	335 (286-367)	338 (293.2-385)	-3.39 (-16.44, 9.65)
**Mean (SD)**	334.9 (69.3)	342.4 (71.2)	
	**Symptom onset to eye exam (days)**		**Mean difference**
**Median (IQR)**	412 (310-528)	470 (332-538.5)	-19.5 (-41.8, 2.76)
**Mean (SD)**	421.4 (119)	447.5 (121)	
	**Percent of acute symptoms reported [3]**		**Mean difference**
**Median (IQR)**	68.75 (56.25-81.25)	68.75 (56.25-81.25)	0.99 (-2.72, 4.71)
**Mean (SD)**	68.62 (18.9)	67.66 (20.1)	
	**Eye symptoms**		**Odds ratio**
**Trouble Seeing**	100 (67.6%)	176 (42.5%)	2.69 (1.78, 4.07)
**Pain in eye**	68 (45.9%)	126 (30.5%)	2.06 (1.39, 3.05)
**Sensitivity to light**	82 (55.4%)	194 (46.9%)	1.43 (0.97, 2.1)
**Redness**	39 (26.4%)	105 (25.4%)	1.01 (0.65, 1.55)
**Discharge**	29 (22.1%)	73 (21.5%)	1.05 (0.64, 1.71)

© 2021 Eghrari AO et al. JAMA Network Open.

	Visual Acuity		Mean difference
Median (IQR)	25 (20-44.72)	20 (20-25)	18.49 (-5.56, 42.55)
Mean (SD)	61 (119)	38 (130)	
	Best Corrected Visual Acuity		Mean difference
Median (IQR)	22.36 (20-32.84)	20 (20-25)	12.57 (-10.36, 35.5)
Mean (SD)	49.61 (98)	34.36 (128)	
	Pinhole visual acuity		Mean difference
Median (IQR)	35.5 (22.36-50)	25 (20-32)	18.87 (-4.81, 42.55)
Mean (SD)	56.14 (81.3)	37.23 (60.6)	
	Color vision (plates)		Mean difference
Median (IQR)	14 (13-14)	14 (13.49-14)	-0.48 (-0.96, 0)
Mean (SD)	12.69 (3.3)	13.26 (2.14)	
	Intraocular pressure (mmHg)		Mean difference
Median (IQR)	11.96 (10-13.96)	12.49 (10.46-14.7)	-0.7 (-1.36, -0.04)
Mean (SD)	12.15 (3.03)	12.86 (3.65)	
	Ocular coherence tomography		Odds Ratio
Mild or greater vitreous opacities	61 (47.3%)	106 (27.6%)	2.2 (1.45, 3.34)
Intraretinal fluid [1]	13 (10.2%)	8 (2.1%)	4.75 (1.88, 11.97)
Epiretinal membrane	9 (11.7%)	6 (2.3%)	5.12 (1.73, 15.16)
Subretinal fluid [1]	2 (50%)	2 (1.6%)	2.51 (0.18, 35.49)
	Central subfield thickness		Odds Ratio
Median (IQR)	222 (210-236)	220.5 (210-233.5)	-1.35 (-6.43, 3.74)
Mean (SD)	222.1 (23.3)	222.6 (19.6)	
	Lab measurements		Odds Ratio
At least 1 positive semen result for Ebola virus RNA[2]	26 (50%)	31 (29.5%)	2.24 (1.12, 4.48)
HIV seropositive [1]	0 (0%)	9 (2.4%)	0 (0, 1.33)

© 2021 Eghrari AO et al. JAMA Network Open.

**Syphilis seropositive**	7 (30.4%)	16 (4.3%)	1.02 (0.4, 2.56)

Supplemental Table 2. Clinical characteristics of Ebola virus disease survivors with and without uveitis. Compared to survivors without uveitis, uveitis patients were more likely to be older, to have a longer stay in the Ebola Treatment Unit, to experience eye pain and trouble seeing, and to have optical coherence tomography findings of mild or greater vitreous opacities, intraretinal fluid, and epiretinal membrane.

© 2021 Eghrari AO et al. JAMA Network Open.

eFigure 1.

Standard optical coherence tomography images viewed by examiners to guide subjective grading of the level of vitreous opacities in the PREVAIL III Eye Substudy. These images are of the eyes of participants from within the local population during the first week of the study.

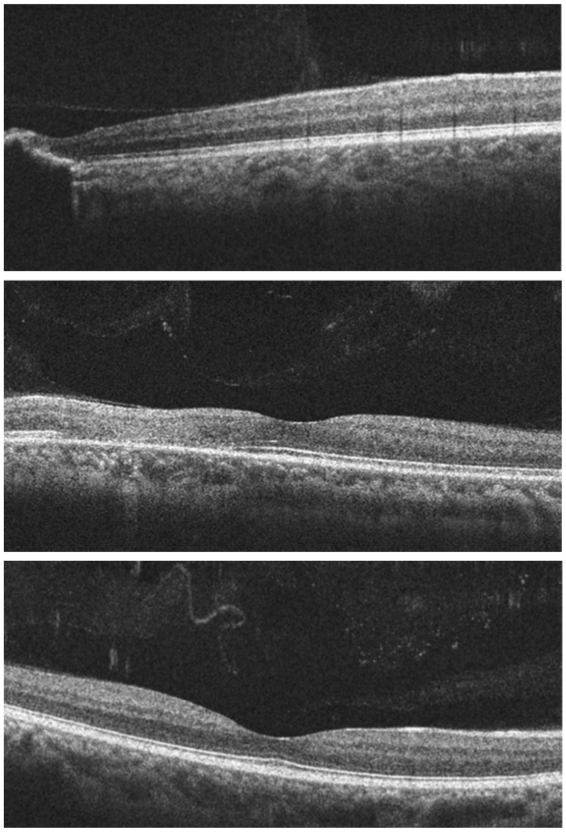

"None to minimal vitreous opacities"

"Mild vitreous opacities"

"Moderate to severe vitreous opacities"

© 2021 Eghrari AO et al. JAMA Network Open.
